# Bilateral Diffuse Tumorous Pseudoangiomatous Stromal Hyperplasia: A Case of Bilateral Mastectomy in a 29-Year-Old Woman

**DOI:** 10.1155/2014/250608

**Published:** 2014-12-02

**Authors:** Hongyan Dai, Carol Connor, Wei Cui, Jason Gatewood, Fang Fan

**Affiliations:** ^1^Department of Pathology and Laboratory Medicine, University of Kansas Medical Center, 3901 Rainbow Boulevard, Kansas City, KS 66160, USA; ^2^Department of Surgery, University of Kansas Medical Center, 3901 Rainbow Boulevard, Kansas City, KS 66160, USA; ^3^Department of Radiology, University of Kansas Medical Center, 3901 Rainbow Boulevard, Kansas City, KS 66160, USA

## Abstract

Pseudoangiomatous stromal hyperplasia (PASH) is a benign breast lesion commonly encountered as an incidental microscopic finding. However, it can also manifest as a mass-forming lesion (tumorous PASH) capable of recurrence after surgical excision. Most of the previously reported cases of tumorous PASH present as a single dominant mass. Here we reported a rare case of diffuse tumorous PASH involving bilateral breasts clinically mimicking malignancy. A 29-year-old African-American female presented with a one-year history of bilateral breast enlargement and asymmetry. Physical examination revealed multiple palpable nodules in bilateral breasts. Imaging studies demonstrated innumerable homogeneously enhancing masses throughout both breasts, greater on the left, with multiple cysts and edema. Biopsy of the breast nodules demonstrated histopathological changes consistent with PASH. Due to the extent of the lesions and progressive clinical symptoms, decision was made to perform bilateral mastectomy. Macroscopic examination of the bilateral mastectomy specimens revealed markedly enlarged breasts with marked edema and numerous well-defined firm nodules. Microscopic evaluation of the nodules confirmed the diagnosis of PASH. No evidence of malignancy was identified. Recognition of this rare form of PASH is essential for the proper clinical management.

## 1. Introduction

Pseudoangiomatous stromal hyperplasia (PASH) is a benign mesenchymal breast lesion characterized histologically by stromal proliferation and presence of anastomosing slit-like spaces lined by bland spindle cells simulating blood vessels [1]. Microfocus of PASH is frequently encountered as an incidental finding in breast specimens and often not reported. Occasionally, PASH can present as a discrete mass or masses (the so-called tumorous PASH) that clinically mimic various benign or malignant conditions [2]. To date, well over 100 cases of tumorous PASH have been described and the majority of these cases report a single well-demarcated mass [3]. Rarely, tumorous PASH can manifest as a diffuse process causing breast enlargement, either unilaterally or bilaterally, with no dominant/discrete mass identified [4, 5]. Here we report a case of symptomatic bilateral diffuse tumorous PASH that occurred in a young female and necessitated bilateral mastectomy.

## 2. Case Report

### 2.1. Clinical Presentation

**Figure 1:**
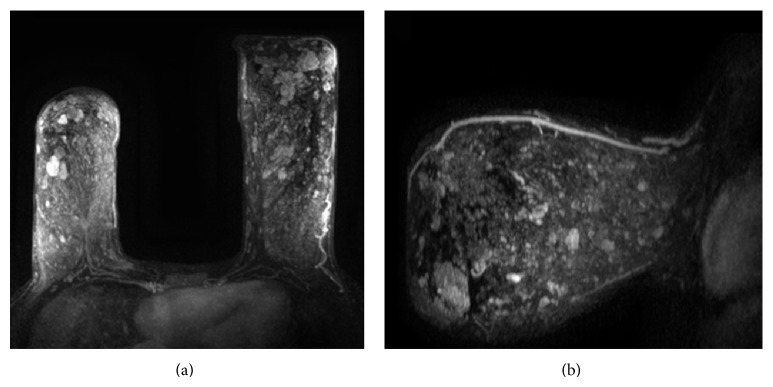
Imaging findings. Contrast enhanced T1 MRI demonstrates innumerable lobulated enhancing masses within both breasts as well as macromastia (a). Sagittal contrast enhanced T1 MRI of the left breast showing macromastia and innumerable lobulated enhancing masses (b).

A previously healthy 29-year-old African-American female presented for evaluation of bilateral progressive breast enlargement, persistent palpable nodules in bilateral breasts, left breast tenderness, left upper extremity pain, and back pain for approximately a year. Previous treatment with antibiotics failed to alleviate symptoms. Physical examination revealed bilateral macromastia, asymmetry, with the left breast being larger than the right, and multiple palpable mammary nodules. Bilateral diagnostic mammograms performed at an outside hospital showed diffuse skin thickening and edema throughout the left breast parenchyma. Left breast sonographic study the same day showed retroareolar ductal prominence with multiple cysts. A skin biopsy was performed by a dermatologist, the result of which was noncontributory. A breast magnetic resonance imaging (MRI) was done at the same outside institution which demonstrated innumerable homogeneously enhancing masses throughout both breasts, greater on the left, with the largest one located on the left measuring up to 3.0 × 2.0 cm. No axillary or internal mammary lymphadenopathy was noted. Differential diagnoses based on imaging studies and clinical presentation included inflammatory breast cancer, severe mastitis, severe fibrocystic changes, and, less likely, phyllodes tumor. The patient reported no family history of breast or ovarian cancer. Two excisional biopsies of the left breast were performed at an outside hospital and were diagnosed as fibroadenoma and lymphangiectasia with associated mild lymphoplasmacytic infiltrate, respectively. The patient was subsequently referred to our institution for further evaluation. The slides of the previous excisional breast biopsies and skin biopsies were obtained and reviewed and were diagnosed as PASH. Sono-guided biopsies of the palpable nodules in the right breast were performed, which revealed fibrosis, duct ectasia, and apocrine metaplasia with no evidence of malignancy. Repeat MRI confirmed the previous findings and demonstrated worsening asymmetry and macromastia (Figure 1). Antihormonal therapy with tamoxifen was recommended; however, the patient declined this treatment option. Due to disease progression, decision was made to perform bilateral mastectomy with immediate reconstruction.

### 2.2. Pathology Findings

**Figure 2:**
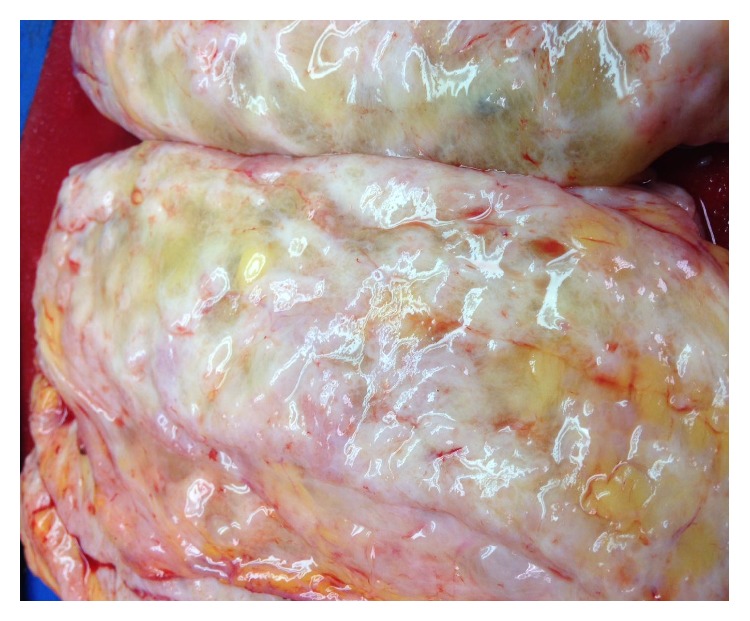
Cross section of the left breast. Numerous tan-pink firm relatively well-demarcated nodules of varying sizes are scattered throughout the breast. Marked edema and poorly formed cysts are also present.

**Figure 3:**
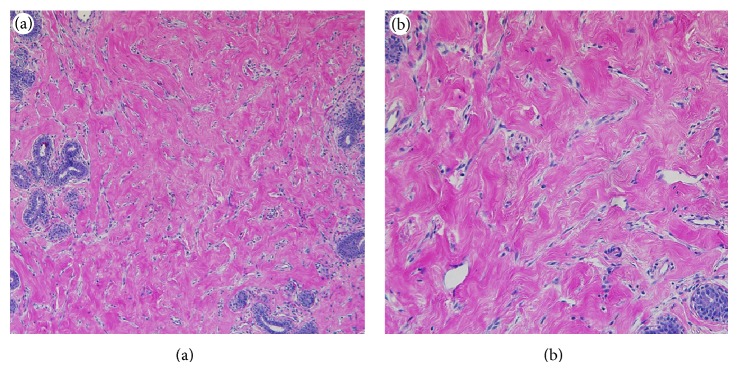
Morphological features of the breast nodules (hematoxylin-eosin stain). Characteristic features of PASH including dense eosinophilic stroma and slit-like spaces lined by attenuated spindle cells are evident (original magnifications ×100 (a) and ×200 (b)).

Left total mastectomy specimen weighed 1770 grams and measured 25.0 × 20.0 × 7.1 cm. Serial sectioning revealed diffuse nodularity, marked edema, and multiple cysts filled with clear fluid (Figure 2). The nodules were rubbery, gray/white in color, and relatively well-circumscribed, with the size of these nodules ranging from 9.0 × 2.7 × 2.0 cm to 0.5 × 0.5 × 0.3 cm. Right total mastectomy specimen weighed 1001 grams and measured 23.0 × 16.8 × 5.2 cm. Serial sectioning of the right breast revealed similar findings. Microscopic examination of the bilateral mammary tissue showed characteristic changes of PASH including stromal hyperplasia and bland spindle cell-lined slit-like open spaces (Figure 3). Additionally, marked stromal edema, dilated lymphatic channels, and mild lymphoplasmacytic infiltrate were also present. Immunohistochemical staining showed that the stromal cells were positive for estrogen receptor (ER) and progesterone receptor (PR) and negative for CD31, supporting the diagnosis of PASH.

## 3. Discussion

Microscopic PASH is a relatively common incidental finding in breast specimens excised for various benign or malignant diagnoses. The exact prevalence of this lesion is unclear. After reviewing 200 consecutive breast specimens, Ibrahim and colleagues [6] reported that at least one microscopic focus of PASH is present in up to 23% of cases. In contrast, mass-forming tumorous PASH is a much rarer entity which is often described in case reports since its first description by Vuitch and colleagues in 1986 [1]. In the present case, we reported a patient with symptomatic bilateral diffuse tumorous PASH which was successfully treated with bilateral simple mastectomy. To the best of our knowledge, only two similar cases have been previously reported in the English literature [4, 5].

Definitive diagnosis of tumorous PASH relies on histopathology evaluation. Majority of these cases manifest as single or, far less frequently, several well-defined benign-appearing masses in female of child bearing age that can be mistaken radiographically and histologically for fibroadenomas, as in the current case. However, in up to 14% of the cases, tumorous PASH can exhibit poorly defined irregular borders on the imaging studies and can be classified as BI-RADS 4 suspicious lesions [2]. Some of these lesions can grow at an alarming speed, raising clinical concerns for malignancy [7–9]. Despite different clinical presentations, the histopathology of tumorous PASH is invariably characterized by dense acellular collagen stroma and slender spindle cell proliferation forming slit-like structures. In the present case, the diagnosis was established one year after patient's initial presentation. Inadequate sampling and unawareness of this disease entity played a significant role in the delay of the diagnosis.

The etiology of PASH is poorly understood. It has been postulated that aberrant response of the myofibroblasts to hormonal effect is at least partially involved in the pathogenesis, a hypothesis which is supported by positive immunohistochemical staining of ER and PR in the majority of cases [10, 11]. This hypothesis also helps to explain the premenopausal age distribution of patients with PASH, although rare cases of PASH have been reported in postmenopausal women and pediatric patients [2, 12]. Lastly, PASH was reported in association with hormone replacement therapy in postmenopausal women and in men with gynecomastia [2, 10, 13]. The above findings strongly suggest an etiological role of sex hormone in the pathogenesis of PASH.

The main differential diagnosis of PASH histologically is low-grade angiosarcoma which is also characterized by anastomosing slit-like channels. However, the slit-like channels in low-grade angiosarcoma are lined by prominent endothelial cells with cytologic atypia. The channels in low-grade angiosarcoma usually contain red blood cells. In contrast, the pseudo-channels in PASH do not contain red blood cells. Low-grade angiosarcoma typically invades the adjacent adipose tissue and distorts the terminal ductal lobular units (TDLU) by involving the intralobular stroma. In PASH, TDLUs are intact and the surrounding adipose tissue is not involved. In difficult cases, immunohistochemical stains are helpful. While both lesions stain positively for CD34, CD31 is usually negative in PASH but positive in angiosarcoma. Additionally, PASH is positive for ER and PR while angiosarcoma is negative for these markers.

Less likely differential diagnoses include fibroadenoma, especially hyalinized fibroadenoma with a pericanalicular pattern, and benign phyllodes tumor. Fibroadenoma has more evenly distributed glandular elements and the stroma does not have the slit-like spaces lined by myofibroblasts. Benign phyllodes tumor has a leaf-like growth pattern and characteristic subepithelial condensation of the stroma. When a tumorous PASH is encountered by a pathologist who is unfamiliar with this entity, it can be misdiagnosed as a fibroadenoma or a benign phyllodes tumor. It has to be noted, however, that PASH can occasionally involve the stromal component of fibroadenoma and phyllodes tumor, creating more challenges for their correct diagnosis.

Management of tumorous PASH is challenging; clinical factors including the size of the lesion, severity of the symptoms, and patients' age have to be all taken into consideration. For incidental focal lesions, no treatment is generally required. For tumorous PASH, surgical excision is commonly performed and proved to be adequate in the majority of cases. Recurrent disease after surgical excision has been reported; whether this is due to incomplete excision or development of a separate lesion is not entirely clear [14]. Given the possible etiological role of estrogen, antihormonal therapy could theoretically serve as an alternative noninvasive approach in the management of tumorous PASH. Successful cases in which patients respond at least partially to tamoxifen therapy have been reported [15, 16]. Undoubtedly, comprehensive clinical study is necessary to evaluate this therapy. As for diffuse tumorous PASH, due to the rarity of this condition, treatment is highly individually based. Breast reduction mammoplasty has been used to manage both unilateral and bilateral diffuse tumorous PASH [4, 5]. In the present case, worsening asymmetry, progressive enlargement of the bilateral breasts, and the patient's refusal of antihormonal therapy necessitated bilateral mastectomy. She has been followed up for 10 months when this paper was prepared and no evidence of recurrent disease has been identified.

In summary, we reported a rare case of symptomatic bilateral diffuse tumorous PASH in a 29-year-old African-American female. Macroscopic inspection of the bilateral mastectomy specimens revealed diffuse nodularity, marked edema, and cysts. Microscopic examination of the nodules revealed characteristic morphological changes of PASH. Recognition of this rare form of PASH is essential for prompt and correct management of these patients.

## References

[1] Vuitch M. F., Rosen P. P., Erlandson R. A. (1986). Pseudoangiomatous hyperplasia of mammary stroma. *Human Pathology*.

[2] Jones K. N., Glazebrook K. N., Reynolds C. (2010). Pseudoangiomatous stromal hyperplasia: imaging findings with pathologic and clinical correlation. *American Journal of Roentgenology*.

[3] Virk R. K., Khan A. (2010). Pseudoangiomatous stromal hyperplasia: an overview. *Archives of Pathology and Laboratory Medicine*.

[4] Sng K. K., Tan S. M., Mancer J. F. K., Tay K. H. (2008). The contrasting presentation and management of pseudoangiomatous stromal hyperplasia of the breast. *Singapore Medical Journal*.

[5] Ryu E. M., Whang I. Y., Chang E. D. (2010). Rapidly growing bilateral pseudoangiomatous stromal hyperplasia of the breast. *Korean Journal of Radiology*.

[6] Ibrahim R. E., Sciotto C. G., Weidner N. (1989). Pseudoangiomatous hyperplasia of mammary stroma. Some observations regarding its clinicopathologic spectrum. *Cancer*.

[7] Teh H.-S., Chiang S.-H., Leung J. W. T., Tan S.-M., Mancer J. F. K. (2007). Rapidly enlarging tumoral pseudoangiomatous stromal hyperplasia in a 15-year-old patient: distinguishing sonographic and magnetic resonance imaging findings and correlation with histologic findings. *Journal of Ultrasound in Medicine*.

[8] Yoo K., Woo O. H., Yong H. S., Kim A., Ryu W. S., Koo B. H., Kang E.-Y. (2007). Fast-growing pseudoangiomatous stromal hyperplasia of the breast: report of a case. *Surgery Today*.

[9] Holloway T. L., Jatoi I. (2013). Tumorous PASH presenting as rapid unilateral breast enlargement. *Mayo Clinic Proceedings*.

[10] Anderson C., Ricci A., Pedersen C. A., Cartun R. W. (1991). Immunocytochemical analysis of estrogen and progesterone receptors in benign stromal lesions of the breast: evidence for hormonal etiology in pseudoangiomatous hyperplasia of mammary stroma. *The American Journal of Surgical Pathology*.

[11] Bowman E., Oprea G., Okoli J., Gundry K., Rizzo M., Gabram-Mendola S., Manne U., Smith G., Pambuccian S., Bumpers H. L. (2012). Pseudoangiomatous stromal hyperplasia (PASH) of the breast: a series of 24 patients. *The Breast Journal*.

[12] Shehata B. M., Fishman I., Collings M. H., Wang J., Poulik J. M., Ricketts R. R., Parker P. M., Heiss K., Bhatia A. M., Worcester H. D., Gow K. W. (2009). Pseudoangiomatous stromal hyperplasia of the breast in pediatric patients: an underrecognized entity. *Pediatric and Developmental Pathology*.

[13] Milanezi M. F., Saggioro F. P., Zanati S. G., Bazan R., Schmitt F. C. (1998). Pseudoangiomatous hyperplasia of mammary stroma associated with gynaecomastia. *Journal of Clinical Pathology*.

[14] Singh K. A., Lewis M. M., Runge R. L., Carlson G. W. (2007). Pseudoangiomatous stromal hyperplasia. A case for bilateral mastectomy in a 12-year-old girl. *Breast Journal*.

[15] Pruthi S., Reynolds C., Johnson R. E., Gisvold J. J. (2001). Tamoxifen in the management of pseudoangiomatous stromal hyperplasia. *The Breast Journal*.

[16] Seltzer M. H., Kintiroglou M. (2003). Pseudoangiomatous hyperplasia and response to tamoxifen therapy. *Breast Journal*.

